# The mechanisms underlying the immune control of Zika virus infection at the maternal-fetal interface

**DOI:** 10.3389/fimmu.2022.1000861

**Published:** 2022-11-22

**Authors:** Ana Espino, Jordi Gouilly, Qian Chen, Philippe Colin, Paul Guerby, Jacques Izopet, Ali Amara, Julie Tabiasco, Reem Al-Daccak, Hicham El Costa, Nabila Jabrane-Ferrat

**Affiliations:** ^1^ Toulouse Institute for Infectious and Inflammatory Diseases (Infinity), CNRS UMR5051, INSERM UMR1291, University of Toulouse III, Toulouse, France; ^2^ Department of Obstetrics and Gynecology, Paule de Viguier Hospital, Toulouse, France; ^3^ Department of Virology, Institut Fédératif de Biologie, Toulouse, France; ^4^ CNRS 7212, INSERM U944, University Paris Cité, Hôpital Saint-Louis, Paris, France; ^5^ INSERM UMRS976, University Paris Cité, Hôpital Saint-Louis, Paris, France

**Keywords:** Zika virus, infection, natural killer cells, maternal-fetal interface, inflammation

## Abstract

Unlike other Flaviviruses, Zika virus (ZIKV) infection during the first trimester of pregnancy causes severe pregnancy outcomes including the devastating microcephaly and diseases associated with placental dysfunctions. We have previously reported that the maternal decidua basalis, the major maternal-fetal interface, serves as a replication platform enabling virus amplification before dissemination to the fetal compartment. However, the rate of congenital infection is quite low, suggesting the presence of a natural barrier against viral infection. Using primary cells from first-trimester pregnancy samples, we investigated in this study how the maternal decidua can interfere with ZIKV infection. Our study reveals that whether through their interactions with dNK cells, the main immune cell population of the first-trimester decidua, or their production of proinflammatory cytokines, decidual stromal cells (DSCs) are the main regulators of ZIKV infection during pregnancy. We also validate the functional role of AXL as a crucial receptor for ZIKV entry in DSCs and demonstrate that targeted inhibition of ligand-receptor interaction at the early stage of the infection is effective in drastically reducing virus pathogenesis at the maternal-fetal interface. Collectively, our results provide insights into the mechanisms through which ZIKV infection and spreading can be limited. The strategy of circumventing viral entry at the maternal-fetus interface limits virus dissemination to fetal tissues, thereby preventing congenital abnormalities.

## Graphical Abstract

BioRender.com.

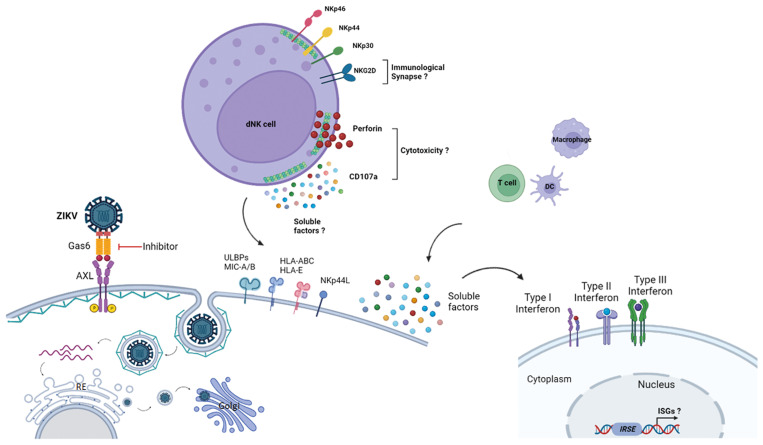

## Introduction

The establishment of immune-privileged fetal-maternal interfaces, including the implantation site, also called decidua basalis (decidua), is a main hallmark of pregnancy. During the first trimester of pregnancy, the maternal decidua hosts a large number of innate lymphoid cells, mainly tissue-resident NK cells and some recruited cells from the peripheral NK cell pool. Decidual NK (dNK) cells account for 70% of leukocytes in the first-trimester decidua (1–3). The majority of these tissue-resident cells are CD56^bright^CD16^neg^ and express most of the activating and inhibitory NK cell receptor repertoire (4–7). During pregnancy, dNK cell responses are finely regulated through interactions with the maternal decidual stromal cells (DSCs), as well as other immune cells and fetal trophoblast cells that invade the maternal decidua. Although dNK cells have functional lytic machinery, but, they are not cytotoxic under healthy conditions. dNK cells produce an array of soluble factors that are mandatory for successful placentation and for the development of the semi-allogenic fetus (8, 9). Yet, dNK cells are also endowed with functional plasticity that allows them to unleash their cytotoxic function and limit the dissemination of pathogens to the fetal compartment (10–12).

Zika Virus (ZIKV) is a single-strand positive-sense RNA virus that belongs to the Flaviviridae family. ZIKV infection manifests with mild symptoms in healthy adults, but it can cause Guillain-Barré syndrome and life-threatening congenital abnormalities referred to as Congenital ZIKV Syndrome in fetuses and offspring when mothers are infected in early pregnancy. Major developmental abnormalities including the devastating microcephaly occur when ZIKV crosses the placenta in early pregnancy, but, the mechanisms involved are far from being fully elucidated (13, 14). Previous reports including ours revealed that ZIKV can replicate in a broad range of maternal and fetal cells from first-trimester placenta such as decidual macrophages and stromal cells, fetal trophoblast cells, and Hofbauer cells as well as mesenchymal stem cells of the umbilical cord (15–18). Nonetheless, maternal-to-fetal transmission is not systematic, emphasizing the effectiveness of the maternal-fetal interface as a natural barrier, and suggesting the existence of efficient antiviral mechanisms during pregnancy.

Although fundamental for developing strategies that prevent maternal-fetal transmission, how ZIKV infection can be contained during pregnancy remains unclear. By using primary cells from the first-trimester pregnancy samples and an ex vivo culture model, we aimed to shed light on this question. Our findings reveal that ZIKV infection shapes the inflammatory environment of the maternal-fetal interface, thereby inducing major phenotypic and functional changes in decidual cells. These changes enabled dNK cells to sense and limit early ZIKV dissemination to fetal cells. Importantly, our results disclose AXL as a main ZIKV entry receptor into the maternal decidua and propose its targeting as an eventual strategy that can prevent congenital infection.

## Results

### ZIKV infects first-trimester maternal decidual stromal cells and alters their NK cell receptor ligand expression

**Figure 1:**
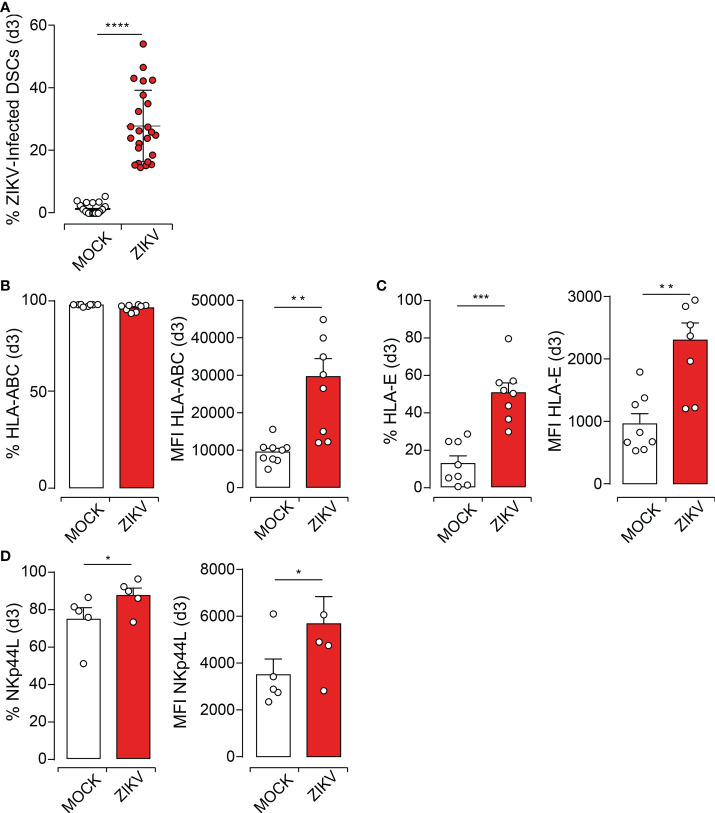
ZIKV Infection of DSCs Modulates the Expression of Key NK Cell Receptor Ligands. **(A)**. DSCs were challenged with ZIKV at an MOI of 1. ZIKV viral protein was determined at day 3 post-infection (dpi) by flow cytometry using anti-NS3 antibody, followed by fluorochrome-conjugated secondary antibody staining. **(B-D)** Cell surface expression of specific NK cell receptor ligands determined at 3dpi by FACS. MOCK (white) and infected (red) DSCs. Percentage and mean fluorescence intensity (MFI) for the expression of HLA-ABC **(B)**, HLA-E molecules **(C)** and NKp44 ligands, NKp44L **(D)**. Data sets represent mean values ± SEM determined from at least five independent donors. *P* values are computed using paired two-tailed Student’s *t* test. *p<0.05, **p<0.01, ***p<0.001, ****p<0.0001.

Previous studies demonstrated that maternal decidua stromal cells (DSCs) are permissive to ZIKV infection (15–18). During pregnancy, dNK cell response is finely regulated through interactions with these cells. Therefore, to determine the potential contribution of dNK cells in controlling ZIKV infection during pregnancy, we first confirmed the ability of the ZIKV Asian strain to infect freshly isolated first-trimester DSCs and determined the optimal conditions for this infection. DSCs were challenged with different multiplicities of infection (MOI of 0.1, 1, and 10). The expression of the Flavivirus envelope group antigen (env) was analyzed by immunofluorescence and confocal microscopy three days post-infection (**Figure S1A**). ZIKV infection was optimal with an MOI of 1. A challenge with a lower MOI of 0.1 was not sufficient, while an MOI of 10 triggered a strong cytopathic effect (**Figure S1A**). These appreciable differences both in terms of viral infection and cytopathic effect prompted us to thereafter carry out all the experiments using this optimal MOI of 1 of the replicative virus to assess the percentage of infected DSCs at different days post-infection (dpi). Analysis of the NS3 viral protein expression by FACS revealed a progressive rising of infection, with approximately 30-60% of infected DSCs at 3 dpi (**Figure 1A** and **Figure S1B**). A slight decline in the number of infected cells was observed at 5 dpi, probably due to the virus cytopathic effect (**Figure S1C**).

Major histocompatibility complex (HLA in humans) class I antigens are expressed in the maternal decidua and play a central role in fine-tuning dNK cell functions (19). Flaviviruses, including ZIKV, are known to interfere with the expression of these HLA antigens as well as with the stress ligands, both considered as major regulators of NK cell function. We, therefore, investigated the impact of ZIKV infection on HLA class I and NK cell receptor ligands expression by DSCs. While the percentage of DSCs constitutively expressing the classical HLA-ABC antigens did not increase, their expression level was significantly increased as evidenced by increased mean fluorescent intensity (MFI) at 3 dpi (**Figure 1B** and **Figure S1D**). Nonetheless, we observed a significant increase in both the percentage and the expression level of the non-classical HLA-E antigens at 3 dpi (**Figure 1C**). Similar results were observed at 5 dpi (**Figures S1D**–**F**).

Although at variable levels, DSCs constitutively express several ligands of NK cell receptors including ligands for NKG2D (NKG2DL), NKp46 (NKp46L), NKp44 (NKp44L), and NKp30 (NKp30L). While ZIKV infection significantly and similarly increased the expression level of NKp44L at 3dpi (**Figure 1D**) and 5dpi (**Figure S1G**), it did not alter the expression of other NK cell receptor ligands such as NKp46L, NKp30L, or NKG2DL (**Figure S2**). Taken together, these results suggest that ZIKV infection increases the levels of some specific stress ligands that could trigger the engagement of NKR and NK cell activation at the maternal-fetal interface.

### Decidual NK cells control early ZIKV infection at the maternal-fetal interface

**Figure 2:**
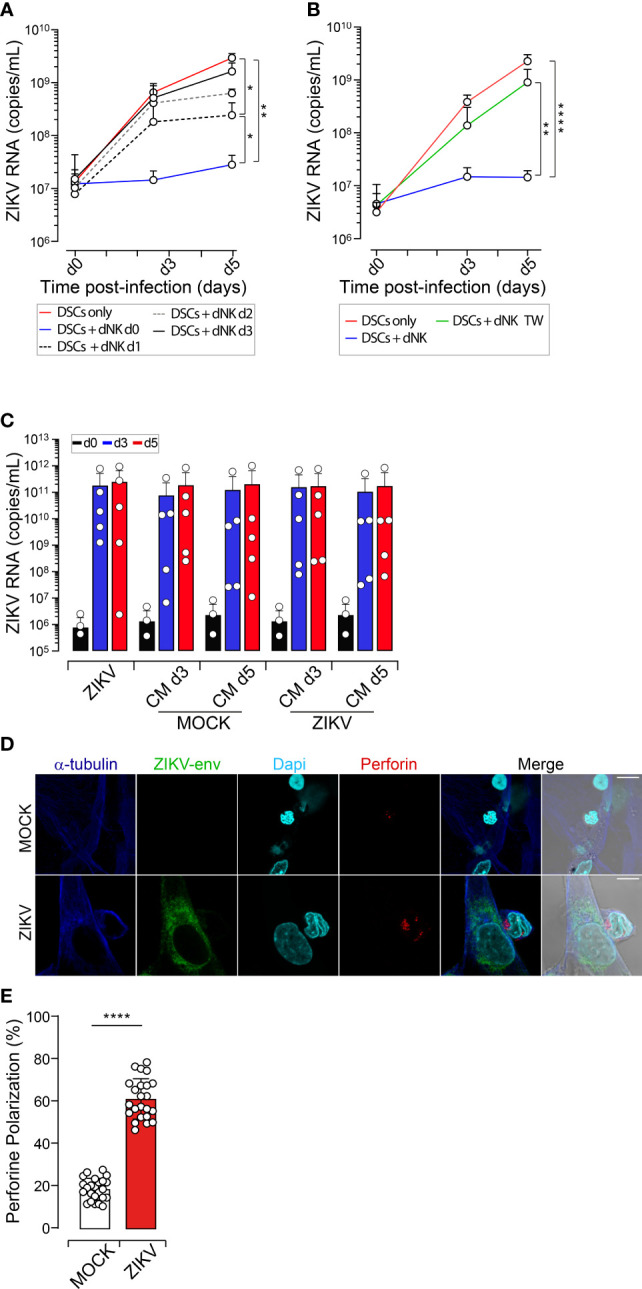
dNK Cells Efficiently Control DSCs Infection. DSCs were infected with ZIKV at an MOI of 1 and co-cultured with autologous dNK cells at a ratio 1:5. **(A-C)** Viral loads are determined by qRT-PCR in cell-free culture supernatants, collected following the indicated time points d0, d3 and d5. **(A)** ZIKV-infected DSCs cultured alone (red line) or with autologous dNK cells added at day 0 (DSCs + dNK d0, blue line), day 1 (DSCs + dNK d1, dotted black line), day 2 (DSCs + dNK d2, dotted grey line) or day 3 (DSCs + dNK d3, solid black line) post-infection. **(B)** ZIKV-infected DSCs cultured alone (DSCs only, red), with autologous dNK cells in the same well (DSCs + dNK, blue) or separately by a 0.4µM porous membrane in transwell system (DSCs + dNK TW, green). **(C)** Fresh DSCs were infected with ZIKV (MOI 1) in the presence of UV-inactivated conditioned media collected from day 3 (CM d3) and day 5 (CM d5) co-cultures of dNK cells with uninfected (Mock) and ZIKV-infected DSCs (ZIKV). Histograms represent quantification of genome replication at day 0 (d0, black), day 3 (d3, blue) and day 5 (d5, red) post-viral challenge. Data sets are presented as mean values ± SEM from at least three independent donors. Statistical significance of differences was evaluated by repeated-measures analysis of variance with the Greenhouse and Geisser correction, and the Newman-Keuls *post hoc* test. *p<0.05, **p<0.01, ****p<0.0001. **(D)** Uninfected and ZIKV-infected DSCs plated on glass coverslips incubated with autologous dNK cells for 20 min at 37°C. Representative confocal images of conjugates with maximum intensity projection are shown. Lytic granules containing perforin (red), ZIKV-env protein (green), α-tubulin (blue) and DAPI nuclei (cyan). Scale bar, 10 µm. **(E)** Percentage of conjugates showing polarized perforin containing granules to the NK cell IS. Results from 5 independent conjugations were averaged. Values represent mean values ± SEM. At least 300 conjugates were analyzed in each experiment (n=3). *P* values are computed using paired two-tailed Student’s t test, ****p<0.0001.

We then investigated within an autologous co-culture model, whether the interaction of dNK cells with ZIKV-infected DSCs could trigger their activation and cytolytic functions to control viral infection. DSCs were infected at an MOI of 1, and co-cultured with purified autologous dNK cells at a 1:5 ratio for 3 and 5 days. Viral replication was monitored over time in culture supernatants by qRT-PCR. After 3 days of co-culture, dNK cells efficiently controlled ZIKV replication in autologous DSCs (**Figure 2A**). The viral load did not increase over time and was similar to the residual 107 RNA copies/mL observed at day 0 post-exposure to ZIKV, compared to infected DSCs cultured in the absence of dNK cells (**Figure 2A**). The control was less efficient when dNK cells were added at a later time post-infection, dropping to less than 50% at 3 dpi and being lost at 5 dpi (**Figure 2A**).

We next determined whether viral control is mediated through direct cell-to-cell contact or the production of soluble mediators. The ability of dNK cells to control viral infection dropped drastically when co-culturing was performed within a double-chamber system that prevented their physical interaction, indicating that the limitation of the infection occurs mainly through cell-to-cell contact (**Figure 2B**). To further confirm, we assessed the capacity of conditioned media (CM) collected from dNK cells-DSCs cocultures to control ZIKV replication. Fresh DSCs were then cultured in UV-inactivated CM before and after exposure to ZIKV inoculum, to avoid infection bias, and viral genome replication was monitored over time. CM also failed to restrict viral replication in DSCs (**Figure 2C**), further confirming that the soluble factors produced by these co-cultures do not play an active role in dNK-mediated control of ZIKV replication.

Cellular contacts between NK cells and their targets occur through the formation of dynamic structures called immune synapses (IS) to deliver the lethal hit through the secretion of lytic granules containing perforin (20). To demonstrate that dNK-mediated control of ZIKV does occur through cell-cell interactions, we, therefore, investigated the capacity of dNK to form IS with autologous ZIKV-infected DSCs. Conjugates formation between dNK cells and uninfected or ZIKV-infected autologous DSCs was analyzed after 20 min of interaction by monitoring perforin localization and polarization to the immune synapse using confocal microscopy. Even though we observed dNK cells forming conjugates with both uninfected and ZIKV-infected target cells, the polarization of perforin to the IS only occurred in conjugates with ZIKV-infected cells (**Figure 2D**). Quantification of perforin polarization demonstrated that while less than 28% of dNK cells showed polarization of their lytic granules engaged in conjugates with uninfected targets, the majority of dNK cells (> 60%) showed polarization of their lytic machinery towards ZIKV-infected DSCs (**Figure 2E**).

Thus, dNK cells in the decidua can efficiently control and limit ZIKV dissemination through direct cellular interactions with infected DSCs. However, this barrier effect only occurs at an early phase of infection.

### Microenvironment of infected decidua contributes to limiting ZIKV dissemination

**Figure 3:**
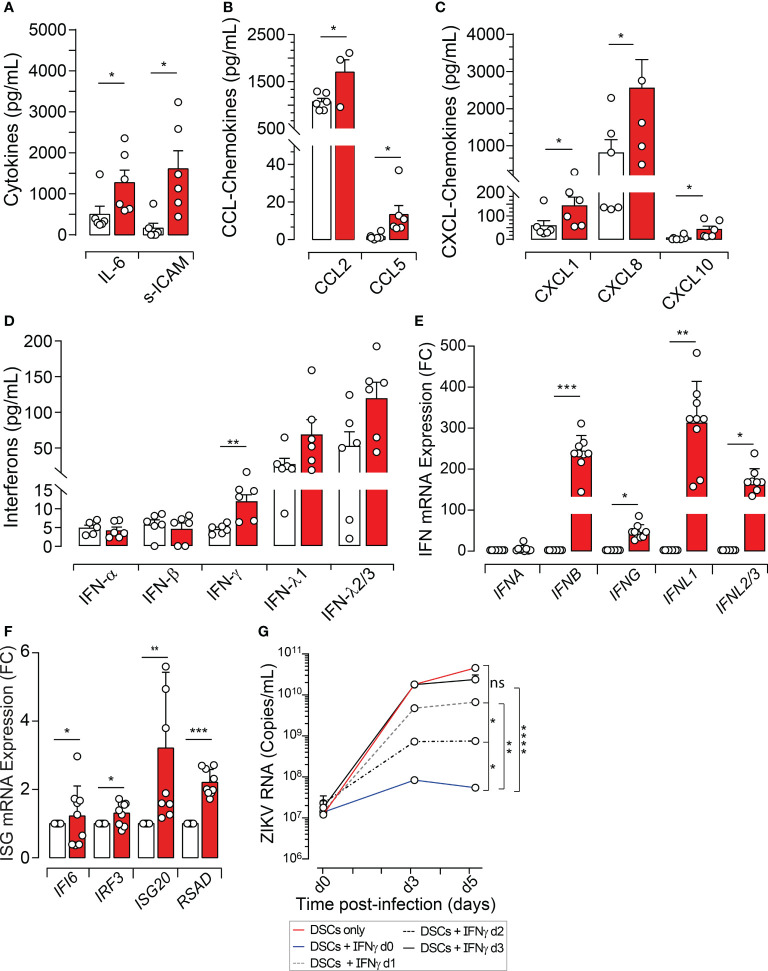
ZIKV Infection Modulates the Decidual Environment and the Interferon Response. DSCs were infected with ZIKV at an MOI of 1. **(A)** Cytokines, **(B)** CCL-chemokines, **(C)** CXCL-chemokines, **(D)** IFN-α, IFN-β, IFN-γ, IFN-λ1 and IFN-λ2/3, quantified in the supernatant using a 42-multi-plexed cytokine assay 48 hours post-infection. **(E, F)** Total mRNA extracted 3 dpi and transcripts quantified using qRT-PCR and specific primers for *IFNA*, *IFNB*, *IFNG*, *IFN*L1, *IFNL2/3*, *IFI6*, *IRF3*, *ISG20* and *RSAD*. Histograms showing significant differences between uninfected (white bars) and ZIKV-infected DSCs (red bars) are presented as fold change of relative mRNA expression (FC). **(G)** ZIKV-infected DSCs cultured with or without recombinant human IFN-γ (100ng/mL). IFN-γ was added at day 0 (d0, blue line), d1 (dotted black line), d2 (dotted grey line) or d3 (black line) post-infection. Non treated ZIKV-infected DSCs (red line) are used as positive control. Culture supernatants were collected from the different conditions following the indicated time points and viral loads were determined by qRT-PCR. All data sets are normalized and presented as mean values ± SEM from at least five independent donors. Statistical significance of differences was evaluated by repeated-measures analysis of variance with the Greenhouse and Geisser correction, and the Newman-Keuls *post hoc* test. ns, not significant. *p<0.05, **p<0.01, ***p<0.001, ****p<0.0001.

Beyond their interaction with dNK cells, DSCs produce various soluble factors and contribute actively to the decidua microenvironment which is mandatory to ensuring immune tolerance of semi-allogenic conceptus, trophoblast attraction, and vasculature remodeling (8). Because our results demonstrated that the contribution of dNK cells to restraining congenital ZIKV infection is limited to early phases of infection, we investigated whether DSCs through miscellaneous secreted factors could contribute to controlling ZIKV propagation during pregnancy. Within this notion, we analyzed the secretion profile of uninfected and ZIKV-infected DSCs. ZIKV infection of DSCs was associated with a significant increase in the level of IL-6, sICAM-1, CCL2, and CCL5 as well as the CXCL1, CXCL8, and CXCL10 chemokines (**Figures 3A-C**), whereas other analytes, either showed variable tendency to increase without reaching significance (IL-1RA, LAP, CCL4, sFasL, TRAIL, M-CSF, and VEGF-A) or did not vary (metalloproteases) upon exposure of DSCs to ZIKV (**Figure S3**).

Importantly, DSC infection by ZIKV also altered the level of the well-known first line of defense against invading pathogen infections, the interferons (IFNs) (21). Namely, ZIKV infection was associated with a significant increase in the level of IFN-γ, a variable tendency to increase the levels of IFN-λ1 and IFN-λ2/3 without reaching statistical significance, but did not genuinely affect the level of either IFN-α or β (**Figure 3D**). These observations prompted us to test whether ZIKV regulates the transcriptional level of IFN genes. As depicted in **Figure 3E**, ZIKV infection significantly increased mRNA levels of *IFNB*, *IFNG*, *IFNL1*, and *IFNL2/3* in DSCs, but, did not affect the transcriptional levels of *IFNA* genes. Because IFNs activate signal transduction cascade(s) leading to the induction of hundreds of interferon-stimulated genes (ISGs), that are critical for controlling viral pathogenesis through a variety of mechanisms (22, 23), we then investigated the effects of ZIKV infection on downstream IFN signaling pathways, by measuring various ISG transcript expressions. We selected a set of model genes (*IRF3*, *IRF7*, *ISG15*, *ISG20*, *MX1*, *RSAD2* (*Viperin*), and *IFI6*) (**Table 1**) and used a qRT-PCR-based expression analysis. In comparison with mock-infected cells, we observed that *IRF3*, *ISG20*, *RSAD2*, and *IFI6* genes were significantly activated at levels ranging from 2- to >4 fold (**Figure 3F**). No significant differences were observed for *IRF7*, *ISG15*, and *MX1* (**Figure S3F**). Thus, our results suggest that ZIKV infection is associated with a robust IFN and ISG response.

**Table 1:** Primer Sequence for Quantitative PCR.

	Primer sequence	
Target Gene	Forward	Reverse
*ZIKV*	TTGGTCATGATACTGCTGATTGC	CCTTCCACAAACTCCCTATTGC
*IFNA*	GAATGAGGACTCCATCCTGGC	TGATTTCTGCTCTGACAACCTCC
	GAATGCGGACTCCATCTTGGC	
	GTACGAGGACTCCATCCTGGC	
	GAAGGAGGACTCCATTCTGGC	
	GAATGTGGACTCCATCCTGGC	
	GAATGAGGACTTCATCCTGGC	
	TGAATGTGGACTCTATCCTGACTG	
*IFNB*	GATACAGCATAGCATCGCCG	ACAATGGCTGGCTCAAGTAGG
*IFNG*	GAGTGTGGAGACCATCAAGGAAG	TGCTTTGCGTTGGACATTCAAGTC
*IFNL1*	GAGGCCCCCAAAAAGGAGTC	AGGTTCCCATCGGCCACATA
*IFNL2/3*	ACACCCTGCACCATATCCTCTC	CGGAAGAGGTTGAAGGTGACAG
*HPRT*	TGACACTGGCAAAACAATGCA	GGTCCTTTTCACCAGCAAGCT
*ISG20*	AGTGAGCGCCTCCTACACAAG	ACCAGCTTGCCTTTCAGGAG
*ISG15*	CGCAGATCACCCAGAAGATCG	TTCGTCGATTTGTCCACCA
*IRF3*	TACCTTCACGGAAGGAAGCG	GCACAACCTTGACCATCACG
*IRF7*	CAGAGCCGTACCTGTCACC	GGGGCCGTATAGGAACGTG
*MX1*	CAGAGAGAAGGAGCTGGAAGAA	GCTGGCCTCCTGGTGATA
*RSAD2*	CAAGACCGGGGAGAATACCTG	GCGAGAATGTCCAAATACTCACC
*IFI6*	AGCTGGTCTGCGATCCTGAATG	TTACCTATGACGACGCTGCTGC

Specific sequences of forward and reverse primers are given for each target gene. To depict different IFNAs, seven degenerated forward primers were designed based on sequence alignments.

To provide a meaningful breadth to DSC response to ZIKV infection, we determined the antiviral potency of IFNs during ZIKV infection. We choose IFN-γ as a representative IFN since it can be produced not only by the DSCs but also by other cells including NK and T cells. ZIKV-infected DSCs were treated with recombinant human IFN-γ, and viral replication was monitored in culture supernatants by qRT-PCR. IFN-γ strongly controlled the infection when DSCs were treated concomitantly to their exposure to ZIKV particles. The inhibition rate of ZIKV genome replication reached almost 95% at 3 dpi and 5 dpi. This control was progressively lost when the antiviral cytokine was added later in the infection (**Figure 3G**).

Taken together, our results demonstrate that ZIKV infection increases the production of key chemokines (CCL2, CCL5, CXCL1, CXCL8, and CXCL10) recognized as a major chemoattractant for various immune cell types to the inflamed or infected sites, but also elicits a robust type II and type III IFN response that can efficiently contribute to controlling early steps of ZIKV infection.

### ZIKV employs AXL receptor to enter the maternal decidua

**Figure 4:**
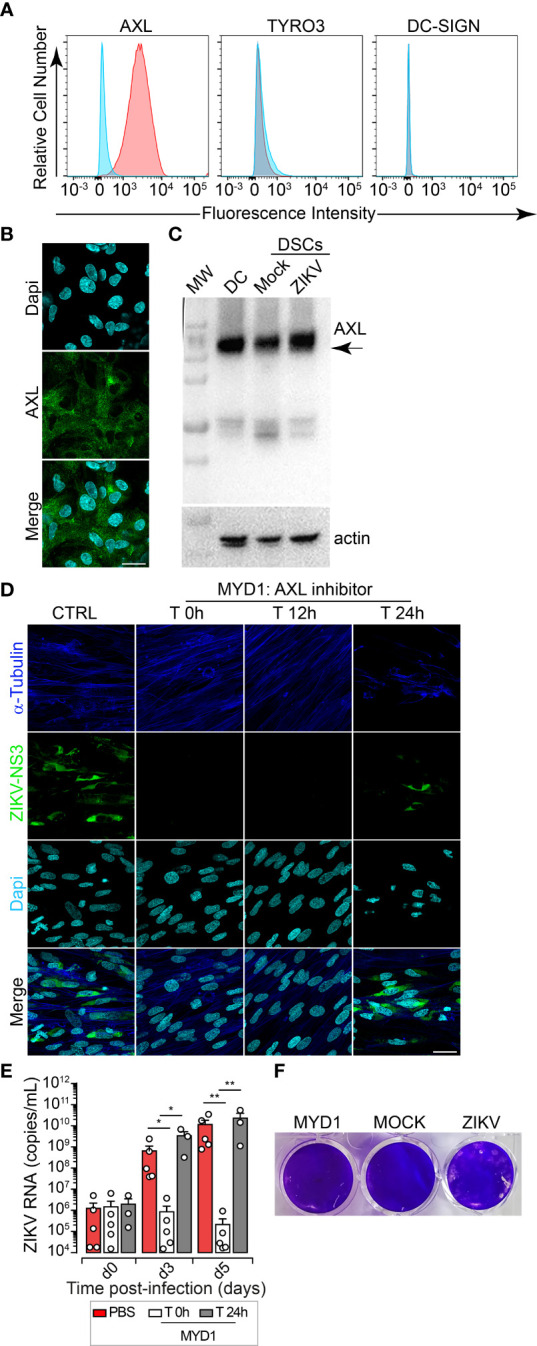
AXL Receptor Is Essential for ZIKV Infection of DSCs. **(A)** Fluorescence immunostaining for cell surface molecules, AXL, TYRO3, DC-SIGN using specific antibodies. Representative histograms gated on live cells are shown. Specific receptor staining (Pink), isotype-matched Ig control (Blue). One representative histogram out of five independent experiments is shown. **(B)** Representative images of maximum intensity projection of DSCs. AXL (green), DAPI nuclei (cyan). Scale bar, 20 µm. Data are representative of at least five independent experiments from five independent donors. **(C)** Western blot analysis of AXL expression. β-actin as the loading control, DC (dendritic cells) expressing AXL protein as positive control. **(D)** ZIKV-DSCs incubated with or without MYD1 decoy receptor, added during infection (T 0h) or 12 hours (T 12h) and 24 hours (T 24h) post-infection. Immunostaining performed 5 dpi in the presence of MYD1 treatment. NS3 (green), α-tubulin (blue) and DAPI nuclei (cyan). Scale bar, 20 µm. Data are representative of at least three independent experiments. **(E)** ZIKV replication in mock treated DSCs (red) and in DCSs that were treated with MYD1 during the infection(white) or 12 hours post-viral challenge (grey). Viral loads were determined by qRT-PCR at days 0,3 and 5 post challenge. Bar graphs represents the mean values ± SEM determined from five independent donors. *P* values are computed using one-way ANOVA with the Dunn *post hoc* test for comparisons of different experimental conditions. *p<0.05, **p<0.01. **(F)** Monolayers were challenged with culture supernatants collected from uninfected DSCs (MOCK) and from cells that were infected with ZIKV in the presence (MYD1) or absence (ZIKV) of MYD1. Plaques were observed at 5 dpi.

Whether through their interactions with dNK or their secreted cytokines, DSCs at the maternal decidua appear as main regulator of ZIKV infection during pregnancy. Therefore, we decided to determine how ZIKV may enter the maternal decidua and DSCs. Several studies have identified DC-SIGN and the TAM (TYRO3, AXL, and MERTK) receptor tyrosine kinase family, which recognizes the externalized phosphatidylserine on apoptotic cells as a natural ligand, as genuine *Flavivirus* receptors (16, 17, 24, 25). We first sought to confirm the expression of the TAM family members (AXL, TYRO3 and DC-SIGN) that could contribute to ZIKV entry in our DSCs model. **Figure 4A** shows that AXL is strongly expressed in DSCs whereas barely few cells, if any, express TYRO3 or DC-SIGN. Immunofluorescence and western blotting analyses further confirmed the expression of AXL and demonstrated that the protein expression level is not affected by ZIKV infection (**Figures 4B, C**).

AXL binds to the externalized phosphatidylserine on apoptotic cells and requires the use of GAS6 (growth arrest-specific gene 6). The resulting receptor kinase activation acts as a pleiotropic antagonist of the innate inflammatory immune response, down-modulating type I interferon signaling pathway, and further facilitating viral infection (26, 27). To validate the role of AXL and explore strategies that might be developed to limit congenital infection, we assessed the effects of an engineered variant of the AXL immunoglobulin-like domain 1, MYD1, which blocks the ligand-receptor interaction through complete neutralization of Gas6 (28, 29). Immunofluorescence using an anti-NS3 antibody demonstrated that binding of MYD1 to GAS6 efficiently inhibits ZIKV entry to DSCs (**Figure 4D**). ZIKV infection was abrogated when the engineered MYD1 is added during the viral challenge and up to 12h post-infection, further confirming the importance of AXL for the viral entry (**Figure 4D**). Viral replication was also monitored over time by qRT-PCR in culture supernatants from five DSCs’ donors. After 3 days of co-culture, we showed efficient control of ZIKV replication with the AXL inhibitor. Indeed, the production of viral RNA was completely blocked when DSCs are challenged with ZIKV virions that were pre-incubated with MYD1 or when the decoy receptor was added during the virus adsorption time (less than 1h) (**Figure 4E**). Similar results were observed after 5 days of co-culture. But, no blockade was observed when MYD1 was added at later time points post-viral entry (24h post-infection). To further confirm the critical role of AXL in ZIKV infection, supernatants were collected from ZIKV-infected DSCs that were treated or not with MYD1, and infectious lytic progeny virions were quantified by the gold standard plaque assay using Vero cells (**Figure 4F**). In agreement with our findings, sequestering GAS6 prevented the production of replication-competent virus particles, compared to the untreated condition.

Altogether, our data validate the functional role of AXL in the pathogenesis of ZIKV at the maternal-fetal interface, acting as a crucial receptor for ZIKV entry in DSCs, and suggest that targeting AXL at an early stage of the infection through the blockade of ligand binding is efficient to drastically reduce ZIKV infection during pregnancy.

## Discussion

Due to the association between ZIKV infection and serious illnesses during pregnancy, both in the infected mother and in the developing fetus, the World Health Organization (WHO) declared ZIKV to be a public health concern (13, 14). The greatest risk for severe pregnancy outcomes with significant fetal neurological malformations has been linked with ZIKV infection in the first trimester of pregnancy. Despite the large tropism of ZIKV at the maternal-fetal interface (15, 17), clinical studies demonstrated that mother-to-fetus transmission only occurs in 4% of the cases, suggesting the existence of antiviral defense mechanisms that limit viral transmission and infection. Within this context, our findings revealed the critical contribution of DSCs and dNK cells in controlling ZIKV replication at the maternal-fetal interface and proposed AXL blockade as an eventual strategy that can limit congenital infection.

Previous investigations demonstrated that viral infections can be associated with dysregulations in the expression of NKR ligands by decidual cells, thereby enhancing the immune response or promoting virus evasion (12, 30). We demonstrated that both classical (HLA-ABC) and non-classical (HLA-E) MHC-I molecules and NKp44L were upregulated by ZIKV. This increase may be essential for viral control but also escape from the immune system since MHC molecules are ligands for both inhibitory and activating receptors of NK cells (31–34), and NKp44 binding to its ligand can mediate both activating and inhibitory signals (35–37). Thus, the control relies on the balance between the inhibitory and the activating signals. Since dNK cells are able to dampen ZIKV replication in DSCs, our results suggest that MHC and/or NKp44L overexpression in ZIKV-infected DSCs triggers the engagement of activating receptors on dNK cells. In agreement with this notion, HLA-E expression on hCMV-infected DSCs was previously shown to bind CD94/NKG2C or CD94/NKG2E activating receptors rather than to the CD94/NKG2A inhibitory receptor (12). Furthermore, NKp44 receptor-ligand binding during West Nile Virus, a member of Flaviviridae family, infection contributes to NK cell activation (38). Interestingly, MHC molecules expression during ZIKV infection seems to be dependent on cell types. Indeed, recently published data showed that HLA-E expression was unchanged in ZIKV-infected placental cell lines (11).

We also found that DSCs constitutively express NKG2DL. Even if we did not detect any changes using Fc-NKG2D chimeric receptor, we cannot exclude the involvement of NKG2D receptor in viral control, since NKG2D can interact with ligands expressed on DSCs and influence the balance between the inhibitory and the activating signals.

Recent reports revealed that dNK cells are able to degranulate and kill ZIKV-infected placental cell lines in part through NKp46 (11). Consistent with these findings, we showed that dNK cells were able to control the replication of ZIKV at the early stages of the infection through the establishment of immune synapse, which is an initial and crucial step for the delivery of lethal hits by NK cells. By contrast, our data suggest the involvement of NKp44 ligand in the formation of immune conjugates with infected cells, but, NKp46 ligand was barely expressed by ZIKV-infected and uninfected DSCs. This discrepancy may be due to cell type and require further analysis. Overall, our findings are in agreement with the previously established notion of dNK cells playing a critical role in the control of maternal viral infection and spreading to the fetus (10–12, 39). However, dNK cells appear to preferentially control ZIKV infection through cytotoxic processes rather than the production of soluble factors. Yet, further studies are warranted to understand the mechanisms at play during immune synapse formation between dNK cells and DSCs. Understanding this mechanism is of major importance, as dNK cell activation and prolonged exposure to lytic molecules can trigger placental immune pathology and contribute to the development of congenital ZIKV infection (40, 41).

Recently published data revealed that ZIKV-infected decidual cells increase transcription of several soluble factors in a gestational age manner (16). In agreement with these observations, we demonstrated here that ZIKV infection of first-trimester DSCs alters the local microenvironment. ZIKV infection was associated with increased secretion of various chemokines (CCL5, CXCL1, CXCL8, CXCL10, and CXCL2) directly linked to the recruitment of immune cells, including T cells, macrophages, and NK cells that are known to actively contribute to the uterine vascular remodeling and the maintenance of fetal tolerance. Chemokines are also essential for trophoblast cell migration and invasion during vascular remodeling as well as for angiogenesis regulation. ZIKV infection also induces IL-6 secretion, most probably through the activation of the IL-6/STAT3 signaling pathway, which plays a key role in regulating inflammatory host immune responses during infections. However, several studies have reported differential regulation of STAT3 in viral infections (42). The STAT3 role in viral replication is complex, as this transcription factor appears to exhibit both pro- or anti-viral functions.

Interferons play a critical role in the antiviral immune response (43, 44), and their production by infected cells can prevent viral propagation. Multiple ZIKV non-structural proteins including NS5 can selectively restrict the innate antiviral responses. By interfering with the RIG-1 and STAT1/2 pathways, ZIKV proteins are able to suppress the antiviral properties of type I and type III interferons while activating type II interferon-triggered signaling cascades (45, 46). In our study, ZIKV infection was associated with the upregulation of IFNB, IFNL, and IFNG at the transcriptional levels, but significant protein production by ZIKV-infected DSCs was only observed for IFN-γ and IFN-γ-stimulated genes such CXCL10. Consistent with our findings, Bowen et al. showed that ZIKV interferes with the production of IFNs by preventing the translation of type I and III IFNs in dendritic cells (47). Such mechanisms may be at play at the maternal-fetal interface and deserve further analysis. The antiviral effect of type II IFN on ZIKV replication is not unanimous. For instance, Chaudhary et al. found that IFN-γ facilitates viral replication in placental cell lines (45). Similar to human skin cells (24), we found that ZIKV replication in DSCs is sensitive to the antiviral effects of type II interferons, suggesting that the anti-viral effects of type II interferon are cell-type dependent. Overall, the production of IFNs may contribute to limiting ZIKV infection during pregnancy but could alter critical steps of placentation (24, 48–53).

We also found that ZIKV infection enhances ISGs production, which is consistent with recent studies demonstrating that viral infections have been associated with the induction of hundreds of ISGs, including OAS2, ISG15 and MX1 (50, 54). ISGs can then contribute to the inhibition of viral replication (22, 23). As a result, IFNs could therefore act as a warning signal to other uninfected cells, thereby preventing viral transmission. Nonetheless, a massive dysregulation of DSC-secreted factors may also have dramatic consequences during the course of pregnancy which may explain, at least in part, why in some cases ZIKV infection is associated with tissue damage (55).

Given the etiology associated with ZIKV infection during pregnancy, the identification of virus entry receptors in decidual cells is critical to understanding the ZIKV pathogenesis and also to developing strategies that prevent congenital infection. While several molecules, including DC-SIGN, AXL, TYRO3, and to a lesser extent TIM-1 permitted ZIKV entry into target cells, the major role was attributed to AXL (17, 24, 25). In addition, previous studies reported a weak mRNA expression of TIM1-4, TYRO3, and MERTK in DSCs (16). In agreement with these reports, our study revealed that AXL is highly expressed in DSCs and is crucial for ZIKV entry in first-trimester DSCs. This observation was further confirmed by using the engineered AXL decoy receptor MYD1, which interferes with the ligand-receptor interaction by sequestering human GAS6 thereby hindering ZIKV entry (56).

In conclusion, our study furthers the knowledge of ZIKV infection during pregnancy by providing new insights into the maternal immune response and defense mechanisms that take place at the maternal-fetal interface. It opens up new perspectives for the development of antiviral strategies to limit the development of the devastating sequelae associated with congenital ZIKV infection.

## Material and methods

### Patient samples and ethics statement

This study was approved by the Research Ethical Comity Haute-Garonne and Agence de Biomédecine (PFS08-022). All the patients included in our study signed the informed consent before samples were taken, in agreement with the Declaration of Helsinki guidelines. All the experiments were performed in accordance with these guidelines.

### Isolation of primary cells

First-trimester tissue *samples* (gestational age 7–12 weeks) were obtained from 18 to 30-year-old healthy women going through elective termination of pregnancy. Decidua samples were processed according to previously described protocols (57). Briefly, minced samples were subjected to collagenase IV (Sigma-Aldrich, France) and DNAseI (Roche) digestion for 45 min at 37 °C under gentle stirring followed by Ficoll-Hypaque density gradient (Amersham Biotech) separation. Cell suspension was then allowed to adhere in tissue culture plates overnight. dNK cells were purified from the non-adherent cell fraction using a CD56 MACS negative selection kit (Miltenyi Biotech). Gating strategies for dNK cells before and after purification are shown in **Figure 4S**. Cell purity was analyzed using a cocktail of antibodies directed to the human CD45, CD14, CD3 and CD56. dNK cells that are CD45^pos^CD3^neg^CD56^pos^ represented at least 70% of the CD45^pos^CD3^neg^CD14^neg^ population. The purity of dNK cells (CD45^pos^CD14^neg^CD3^neg^CD56^pos^) reached 99% after purification using CD56 MACS negative selection kit (**Figure S4B**). dNK cells were kept at 4°C in conditioned media containing 20% heat-inactivated fetal calf serum (FCS) for subsequent experiments. DSCs were isolated from the adherent fraction by successive rounds of mild trypsin treatment and processed for subsequent experiments. DSCs (Cytokeratin 7^neg^Vimentin^pos^ CD14^neg^) were maintained in DMEM:F12 (v:v) culture medium (Invitrogen) supplemented with 10% FCS and penicillin-streptomycin 100 U/mL each, under a 5% CO_2_ atmosphere at 37°C.

### Isolation and propagation of ZIKV

ZIKV was isolated from sperm sample of immunocompetent Caucasian patient returning from Brazil (55). The patient was tested HIV negative and potential infection with dengue or chikungunya viruses were ruled by ELISA Diapro (Diagnostic Bioprobes Srl, https://www.diapro.it) and RealStar Dengue and Chikungunya qRT-PCR (Altona Diagnostics, https://www.altona-diagnostics.com). Phylogenetic analysis of a 1079-nt fragment within the NS5 gene have confirmed that the imported ZIKV strain belongs to the Asian lineage H/PF/2013 (GenBank accession numbers KU886298.1). High titer stocks were obtained from early virus passaging in Vero cells (Vero ATCC^®^ CCL-81™). Briefly, the infected patient’ semen sample was used to infect Vero cells. High titer supernatants were collected from 5 days’ post-infection (dpi). Viral titers were determined by RT-PCR and plaque assays on Vero cells. High titer viral stocks were stored as single use-aliquots and stored at −80 °C.

### Primary cells infection and cultures

Primary DSCs were mock or ZIKV infected overnight with ZIKV particles at the indicated multiplicity of infection (MOI) in DMEM:F12 containing 2% FCS. After several washes in PBS, primary cells were cultured in DMEM:F12 10% FCS-medium for one to five days, depending on the experiment.

For co-culture assays, DSCs were cultured with dNK cells at a 1:5 ratio either through direct co-culture or through double-chamber co-culture system (Corning Incorporated Costar). dNK cells were added at different times post-infection.

### Preparation of conditioned medium

For some conditions, culture supernatants were collected from mock- or ZIKV-infected DSCs-dNK cell co-cultures at day 3 and 5 post-infection. Collected conditioned media (CM) were UV-irradiated for 30 min using Spectroline EF-140/F UV lamp (220 volts, 50 HZ, 17 Amps) to inactivate viral replication. Fresh DSC cultures were then challenged with UV-irradiated CM for 3 or 5 days before further analyses. Non-UV-treated replicative virus inoculum was used as a positive control.

In some experiments, ZIKV-infected DSCs were cultured in the presence of IFN-γ (100ng/mL, R&D System), which was added either during the inoculation or at different time post-infection. ZIKV-infected DSCs were also treated with an AXL decoy receptor (MYD1, 1:1,000 dilution) as previously described (29), added either before infection, as a pre-treatment, or after infection. Culture supernatants were finally collected and either stored at -80°C or used directly for subsequent analysis.

### Quantification of ZIKV RNA

Viral RNA was extracted from 140 µL of culture supernatants collected at different dpi using QIAmp Viral RNA extraction kits (Qiagen). Purified RNA was then reverse transcribed using the SuperScript III first-strand synthesis system kit (Invitrogen). ZIKV genome was detected by quantitative RT-PCR amplification of an NS5 fragment using in house primers (**Table 1**) and a Light Cycler 480 instrument (Roche Molecular Systems) according to the manufacturer’s instructions. Internal standards were included to correct for potential variations in the amount of input material. Quantitative RT-PCR (qRT-PCR) values were further normalized and are given as viral RNA copy numbers/mL. The kinetics of viral genome replication was determined as RNA copy numbers/mL, calculated based on a standard curve.

### Quantification of gene expression by qRT-PCR

Total RNA from MOCK- or ZIKV-infected samples were extracted using the RNeasy Kit (Qiagen) according to the manufacturer’s instructions. Purified RNA was subjected to a reverse transcription using the SuperScript III first-strand synthesis system kit (Invitrogen). Gene expression was quantified by qRT-PCR using Light Cycler 480 SYBR green I master mix (Roche) and specific primers (**Table 1**). qRT-PCR were performed in 96-well plates and run on a Light Cycler 480 instrument (Roche). The housekeeping hypoxanthine-guanine phosphoribosyl transferase (*HPRT*) was used as control. The results are presented as fold change from matched mock-infected controls calculated using the 2^−ΔΔCT^ method.

### Flow cytometry analysis

Cell were washed with PBS and stained with the LIVE/DEAD™ Fixable Yellow Dead Cell Stain Kit, according to the manufacturer’s procedure (*In vivo*gen). 5x10^5^ live cells were used for each condition/experiment. The following antibodies CD3-Vioblue, CD14-FITC, CD56-APC (Miltenyi), CD4-PE, CD8-PerCp, CD45-PeCy7 (Sysmex) were used to characterize dNK cells. The expression of NCR-ligands on DSCs was analyzed using specific Receptor-Fc chimera followed by immunostaining with the APC-coupled mouse anti-human IgG1 secondary Ab (R&D System, France). The following chimeras were used: NKp30-Fc, NKp44-Fc, NKp46-Fc and NKG2D-Fc (R&D Systems, France). The expression of the major histocompatibility complex (MHC) was measured using an anti-human HLA-ABC-PE (BD Pharmingen) and HLA-E-APC (Miltenyi). AXL and TYRO3 expression was determined using the anti-AXL (7E10, Abcam) and anti-TYRO3 (OTI5B7, Abcam) antibodies flowed by immunostaining with class-specific fluorochrome-conjugated secondary antibodies. The anti-CD209-APC-Cy7 (Miltenyi) was used to analyse the expression of DC-SIGN. Samples subjected to immunostaining were analyzed on a BD LSR-FORTESSA cytometer. Acquired data were further analyzed using FlowJo™ software 10.1. Cell staining was depicted as percentage of expression (%) and/or mean fluorescent intensity (MFI) that refer to the proportion of cells expressing a given marker and its level/density of expression respectively.

### Plaque assay

Culture supernatants were collected from ZIKV-infected cells at the indicated time post-infection. Confluent monolayer of Vero cells grown in 12-well plates is challenged with serial dilutions of unknown starting concentration virus-containing supernatants. After adsorption and washing of excess supernatants, the cell layer is overlaid with 2% low-melting agarose (Invitrogen) in 1% FBS DMEM solution, to prevent virus spread and confine virus growth to foci of initially infected cells. After 5 days of incubation, foci develop into plaques. Staining with crystal violet enhances the contrast between plaques and the uninfected monolayer of cells. Plaques are then enumerated and used to determine the titer of infectious progenies in the supernatant as plaque-forming units per mL (PFU/mL).

### Fluorescent microscopy

ZIKV-infected or uninfected DSCs were seeded into 24-well plates containing 0.12 mm glass coverslips. For immune synapse analysis, dNK cells were added to each well of ZIKV-infected or uninfected DSCs plated on glass-cover slips at a 1 to 5 ratio and incubated at 37°C. Conjugates were allowed to form for 20 min at 37°C. Coverslips were gently washed with PBS, fixed with 4% paraformaldehyde (PFA) for 20 min, and permeabilized with PBS 0.3% Triton X-100.

The following primary antibodies were used to analyze ZIKV infection, cell structure, and polarization of lytic granules: anti-human α-Tubulin (DM1A, 1:250 dilution, Sigma-Aldrich, UK), anti-human perforin (1:250 dilution, BD Biosciences Pharmingen) and anti-Flavivirus group antigen (D1-4G2-4-15, 1:400 dilution, Merck-Millipore MAB10216) anti-Dengue virus NS3 (1:100 dilution, abm). Immunostaining was performed at 4 °C overnight. Antigen staining was visualized with Alexa fluor-conjugated 633 (AF-633), 555 (AF-555), and 488 (AF-488) class-specific secondary antibodies (1:400 dilution, Invitrogen). Nuclei were visualized with 4,6-diamidino-2-phenylindole (Dapi, 1:10000 dilution, Sigma) staining.

Large field microscopy was performed with Leica DM4000B microscope (Leica, Solms, Germany, LAS version 3.7.0). Confocal z-stacks were captured using Zeiss LSM710 confocal microscope (Carl Zeiss AG, Jena, Germany). 10×, 20×, or 63× oil objectives were used for all acquisitions. Images were processed using Imaris software (Bitplane AG, Switzerland) and Zen software to obtain maximum intensity projection along the z-axis. The quantification values of infection were obtained by calculating the percentage of infected cells over the total cells for each view field.

To quantify perforin polarization to the synaptic area a minimum of 300 conjugates were analyzed and data are presented as a percentage of polarized immune synapses formed with infected and uninfected DSCs.

### Western blotting

5x10^6^ cells were lysed in RIPA buffer in the presence of a protease inhibitor cocktail (ThermoFisher) for 30 min at 4°C. Protein concentration was determined by Bradford assay (ThermoFisher). Dendritic cells (DC) were derived from CD14^pos^ peripheral blood monocytes (CD14 positive selection kit, Miltenyi) that were cultured for five days with 20 ng/ml recombinant GM-CSF (Miltenyi) and 50 ng/ml IL-13 (Sanofi-Aventis donation). Equal amounts of protein were resolved on 4-20% Mini Protean gradient gels (Bio-Rad Laboratories) then transferred to nitrocellulose membranes (ThermoFisher). Protein expression was analyzed using anti-AXL (C89E7, Cell Signaling Technology) or anti-DC-SIGN (D7F5C, Cell Signaling Technology) rabbit mAbs followed by HRP-conjugated Goat Anti-Rabbit IgG secondary antibody (Jackson ImmunoResearch). Blots were developed by chemiluminescence assay (Bio-Rad Laboratories). Anti-β-actin (mouse mAb, Sigma Aldrich) was used to confirm protein loading.

### Multiplex cytokine and chemokine arrays

Cell free culture supernatants from 5 independent donors’ experiments were collected 48 hours’ post-infection and stored at −80 °C for quantification of soluble mediators. Cytokines, chemokines and growth factors levels were measured using a multiplexed Affymetrix cytokine assay according to the manufacturer protocol (Procarta/Ozyme). The following cytokines and chemokines were analyzed: IL-1β, IL-1, IL-6, CXCL8, IL-10, IL-12, IL-1RA, TNF-α, IFN-α, IFN-β, IFN-γ, IFN-λ, CCL2, CCL3, CCL4, CCL5, CXCL1, CXCL10, CXCL12, G-CSF, GM-CSF, M-CSF, FGF2, VEGF-A, sFasL, sICAM-1, TRAIL, Granzyme B, MMP-2, MMP-9 and LAP. Measurement and analysis were performed using the BioRad Bio-Plex System (BioRad, France).

### Quantification and statistical analysis

All experiments were conducted on donor matched DSCs and dNK cells. At least three independent donors were used for experiments. Statistical analyses were performed using the GraphPad Prism software 9 (GraphPad Software, La Jolla, USA). Graphs represent mean values ± S.E.M. *P* values were determined by two-tailed Student’s t test to compare two parameters. For paired and unpaired multiple comparisons, statistical significance of differences was evaluated by repeated-measures analysis of variance with the Greenhouse and Geisser correction, and the Dunn or Newman-Keuls *post hoc* test. *P*-values of less than 0.05 are considered statistically significant and shown for each Figure. *p < 0.05, **p < 0.01, ***p < 0.001, ****p<0.0001.

## Data availability statement

The original contributions presented in the study are included in the article/**Supplementary Material**. Further inquiries can be directed to the corresponding author.

## Author contributions

AE and JG conducted the experiments, analyzed data and drafted the manuscript; QC, PC, HC and JT contributed to data analysis; PG provided clinical samples; AA provided AXL inhibitor; JI provided financial support and critical discussion of the manuscript; NJ-F designed and supervised the research; RA-D, HC and NJ-F revised and approved the paper. All authors contributed to the article and approved the submitted version.

## Funding

This work was supported by funds from the INSERM-CNRS-University Toulouse III, the Région Occitanie and the Fondation pour la Recherche Médicale (FRM).

## Acknowledgments

We are grateful to the clinical staff from Paule de Viguier maternity hospital for placenta collection and, to the cell imaging and cytometry core facilities for technical assistance. We thank Dr. Ray Tabibiazar, Ruga corporation, Houston, TX 77010-1018, USA) for providing the AXl inhibitor and Prof E. Bahraoui for critical comments on the manuscript. English language usage, grammar, punctuation and spelling by Ms. Dinah McCarthy (mccarthydinah@gmail.com).

## Conflict of interest

The authors declare that the research was conducted in the absence of any commercial or financial relationships that could be construed as a potential conflict of interest.

## Publisher’s note

All claims expressed in this article are solely those of the authors and do not necessarily represent those of their affiliated organizations, or those of the publisher, the editors and the reviewers. Any product that may be evaluated in this article, or claim that may be made by its manufacturer, is not guaranteed or endorsed by the publisher.

## References

[1] Manaster I Mandelboim O. The unique properties of uterine NK cells. Am J Reprod Immunol (2010) 63(6):434–44. doi: 10.1111/j.1600-0897.2009.00794.x 20055791

[2] Moffett A Loke C. Immunology of placentation in eutherian mammals. Nat Rev Immunol (2006) 6(8):584–94. doi: 10.1038/nri1897 16868549

[3] Whitelaw PF Croy BA. Granulated lymphocytes of pregnancy. Placenta (1996) 17(8):533–43. doi: 10.1016/s0143-4004(96)80070-1 8916201

[4] El Costa H Casemayou A Aguerre-Girr M Rabot M Berrebi A Parant O. Critical and differential roles of NKp46- and NKp30-activating receptors expressed by uterine NK cells in early pregnancy. J Immunol (2008) 181(5):3009–17. doi: 10.4049/jimmunol.181.5.3009 18713971

[5] El Costa H Tabiasco J Berrebi A Parant O Aguerre-Girr M Piccinni MP. Effector functions of human decidual NK cells in healthy early pregnancy are dependent on the specific engagement of natural cytotoxicity receptors. J Reprod Immunol (2009) 82(2):142–7. doi: 10.1016/j.jri.2009.06.123 19615756

[6] Jabrane-Ferrat N Siewiera J. The up side of decidual natural killer cells: new developments in immunology of pregnancy. Immunology (2014) 141(4):490–7. doi: 10.1111/imm.12218 PMC3956423 24256296

[7] Male V Sharkey A Masters L Kennedy PR Farrell LE Moffett A. The effect of pregnancy on the uterine NK cell KIR repertoire. Eur J Immunol (2011) 41(10):3017–27. doi: 10.1002/eji.201141445 PMC3262970 21739430

[8] Hanna J Goldman-Wohl D Hamani Y Avraham I Greenfield C Natanson-Yaron S. Decidual NK cells regulate key developmental processes at the human fetal-maternal interface. Nat Med (2006) 12(9):1065–74. doi: 10.1038/nm1452 16892062

[9] Moffett A Colucci F. Uterine NK cells: active regulators at the maternal-fetal interface. J Clin Invest (2014) 124(5):1872–9. doi: 10.1172/JCI68107 PMC4001528 24789879

[10] Crespo AC Mulik S Dotiwala F Ansara JA Sen Santara S Ingersoll K. Decidual NK cells transfer granulysin to selectively kill bacteria in trophoblasts. Cell (2020) 182(5):1125–39.e18. doi: 10.1016/j.cell.2020.07.019 32822574 PMC7484179

[11] Sen Santara S Crespo AC Mulik S Ovies C Boulenouar S Strominger JL. Decidual NK cells kill zika virus-infected trophoblasts. Proc Natl Acad Sci U.S.A. (2021) 118(47). doi: 10.1073/pnas.2115410118 PMC8617421 34785597

[12] Siewiera J El Costa H Tabiasco J Berrebi A Cartron G Le Bouteiller P. Human cytomegalovirus infection elicits new decidual natural killer cell effector functions. PloS Pathog (2013) 9(4):e1003257. doi: 10.1371/journal.ppat.1003257 23592985 PMC3617138

[13] Brasil P Pereira JP Jr. Moreira ME Ribeiro Nogueira RM Damasceno L Wakimoto M. Zika virus infection in pregnant women in Rio de Janeiro. N Engl J Med (2016) 375(24):2321–34. doi: 10.1056/NEJMoa1602412 PMC5323261 26943629

[14] Mlakar J Korva M Tul N Popovic M Poljsak-Prijatelj M Mraz J. Zika virus associated with microcephaly. N Engl J Med (2016) 374(10):951–8. doi: 10.1056/NEJMoa1600651 26862926

[15] El Costa H Gouilly J Mansuy JM Chen Q Levy C Cartron G. ZIKA virus reveals broad tissue and cell tropism during the first trimester of pregnancy. Sci Rep (2016) 6:35296. doi: 10.1038/srep35296 27759009 PMC5069472

[16] Guzeloglu-Kayisli O Guo X Tang Z Semerci N Ozmen A Larsen K. Zika virus-infected decidual cells elicit a gestational age-dependent innate immune response and exaggerate trophoblast zika permissiveness: Implication for vertical transmission. J Immunol (2020) 205(11):3083–94. doi: 10.4049/jimmunol.2000713 33139490

[17] Tabata T Petitt M Puerta-Guardo H Michlmayr D Wang C Fang-Hoover J. Zika virus targets different primary human placental cells, suggesting two routes for vertical transmission. Cell Host Microbe (2016) 20(2):155–66. doi: 10.1016/j.chom.2016.07.002 PMC5257282 27443522

[18] Weisblum Y Oiknine-Djian E Vorontsov OM Haimov-Kochman R Zakay-Rones Z Meir K. Zika virus infects early- and midgestation human maternal decidual tissues, inducing distinct innate tissue responses in the maternal-fetal interface. J Virol (2017) 91(4). doi: 10.1128/JVI.01905-16 PMC5286880 27974560

[19] Moffett-King A. Natural killer cells and pregnancy. Nat Rev Immunol (2002) 2(9):656–63. doi: 10.1038/nri886 12209134

[20] Orange JS. Formation and function of the lytic NK-cell immunological synapse. Nat Rev Immunol (2008) 8(9):713–25. doi: 10.1038/nri2381 PMC2772177 19172692

[21] Carty M Guy C Bowie AG. Detection of viral infections by innate immunity. Biochem Pharmacol (2021) 183:114316. doi: 10.1016/j.bcp.2020.114316 33152343

[22] Schoggins JW. Interferon-stimulated genes: roles in viral pathogenesis. Curr Opin Virol (2014) 6:40–6. doi: 10.1016/j.coviro.2014.03.006 PMC4077717 24713352

[23] Schoggins JW. Interferon-stimulated genes: What do they all do? Annu Rev Virol (2019) 6(1):567–84. doi: 10.1146/annurev-virology-092818-015756 31283436

[24] Hamel R Dejarnac O Wichit S Ekchariyawat P Neyret A Luplertlop N. Biology of zika virus infection in human skin cells. J Virol (2015) 89(17):8880–96. doi: 10.1128/JVI.00354-15 PMC4524089 26085147

[25] Perera-Lecoin M Meertens L Carnec X Amara A. Flavivirus entry receptors: an update. Viruses (2013) 6(1):69–88. doi: 10.3390/v6010069 24381034 PMC3917432

[26] Anderson HA Maylock CA Williams JA Paweletz CP Shu H Shacter E. Serum-derived protein s binds to phosphatidylserine and stimulates the phagocytosis of apoptotic cells. Nat Immunol (2003) 4(1):87–91. doi: 10.1038/ni871 12447359

[27] Rothlin CV Ghosh S Zuniga EI Oldstone MB Lemke G. TAM receptors are pleiotropic inhibitors of the innate immune response. Cell (2007) 131(6):1124–36. doi: 10.1016/j.cell.2007.10.034 18083102

[28] Kariolis MS Miao YR Jones DS 2nd Kapur S Mathews II Giaccia AJ. An engineered axl 'decoy receptor' effectively silences the Gas6-axl signaling axis. Nat Chem Biol (2014) 10(11):977–83. doi: 10.1038/nchembio.1636 PMC4372605 25242553

[29] Meertens L Labeau A Dejarnac O Cipriani S Sinigaglia L Bonnet-Madin L. Axl mediates ZIKA virus entry in human glial cells and modulates innate immune responses. Cell Rep (2017) 18(2):324–33. doi: 10.1016/j.celrep.2016.12.045 28076778

[30] Golden-Mason L Cox AL Randall JA Cheng L Rosen HR. Increased natural killer cell cytotoxicity and NKp30 expression protects against hepatitis c virus infection in high-risk individuals and inhibits replication *in vitro* . Hepatology (2010) 52(5):1581–9. doi: 10.1002/hep.23896 PMC2967665 20812318

[31] Braud VM Allan DS O'Callaghan CA Soderstrom K D'Andrea A Ogg GS. HLA-e binds to natural killer cell receptors CD94/NKG2A, b and c. Nature (1998) 391(6669):795–9. doi: 10.1038/35869 9486650

[32] Kaiser BK Barahmand-Pour F Paulsene W Medley S Geraghty DE Strong RK. Interactions between NKG2x immunoreceptors and HLA-e ligands display overlapping affinities and thermodynamics. J Immunol (2005) 174(5):2878–84. doi: 10.4049/jimmunol.174.5.2878 15728498

[33] Jun Y Kim E Jin M Sung HC Han H Geraghty DE. Human cytomegalovirus gene products US3 and US6 down-regulate trophoblast class I MHC molecules. J Immunol (2000) 164(2):805–11. doi: 10.4049/jimmunol.164.2.805 10623826

[34] Nattermann J Nischalke HD Hofmeister V Kupfer B Ahlenstiel G Feldmann G. HIV-1 infection leads to increased HLA-e expression resulting in impaired function of natural killer cells. Antivir Ther (2005) 10(1):95–107. doi: 10.1177/135965350501000107 15751767

[35] Bruhns P Marchetti P Fridman WH Vivier E Daeron M. Differential roles of n- and c-terminal immunoreceptor tyrosine-based inhibition motifs during inhibition of cell activation by killer cell inhibitory receptors. J Immunol (1999) 162(6):3168–75. 10092767

[36] Lanier LL Corliss BC Wu J Leong C Phillips JH. Immunoreceptor DAP12 bearing a tyrosine-based activation motif is involved in activating NK cells. Nature (1998) 391(6668):703–7. doi: 10.1038/35642 9490415

[37] Wu J Cherwinski H Spies T Phillips JH Lanier LL. DAP10 and DAP12 form distinct, but functionally cooperative, receptor complexes in natural killer cells. J Exp Med (2000) 192(7):1059–68. doi: 10.1084/jem.192.7.1059 PMC2193316 11015446

[38] Hershkovitz O Rosental B Rosenberg LA Navarro-Sanchez ME Jivov S Zilka A. NKp44 receptor mediates interaction of the envelope glycoproteins from the West Nile and dengue viruses with NK cells. J Immunol (2009) 183(4):2610–21. doi: 10.4049/jimmunol.0802806 PMC2768489 19635919

[39] Quillay H El Costa H Duriez M Marlin R Cannou C Madec Y. NK cells control HIV-1 infection of macrophages through soluble factors and cellular contacts in the human decidua. Retrovirology (2016) 13(1):39. doi: 10.1186/s12977-016-0271-z 27267272 PMC4895978

[40] Szekeres-Bartho J. Regulation of NK cell cytotoxicity during pregnancy. Reprod BioMed Online (2008) 16(2):211–7. doi: 10.1016/s1472-6483(10)60576-7 18284875

[41] Zhang X Wei H. Role of decidual natural killer cells in human pregnancy and related pregnancy complications. Front Immunol (2021) 12:728291. doi: 10.3389/fimmu.2021.728291 34512661 PMC8426434

[42] Kuchipudi SV. The complex role of STAT3 in viral infections. J Immunol Res (2015) 2015:272359. doi: 10.1155/2015/272359 26199948 PMC4496485

[43] Ngono AE Shresta S. Immune response to dengue and zika. Annu Rev Immunol (2018) 36:279–308. doi: 10.1146/annurev-immunol-042617-053142 29345964 PMC5910217

[44] Samuel CE. Antiviral actions of interferons. Clin Microbiol Rev (2001) 14(4):778–809. doi: 10.1128/CMR.14.4.778-809.2001 11585785 PMC89003

[45] Chaudhary V Yuen KS Chan JF Chan CP Wang PH Cai JP. Selective activation of type II interferon signaling by zika virus NS5 protein. J Virol (2017) 91(14). doi: 10.1128/JVI.00163-17 PMC5487581 28468880

[46] Lundberg R Melen K Westenius V Jiang M Osterlund P Khan H. Zika virus non-structural protein NS5 inhibits the RIG-I pathway and interferon lambda 1 promoter activation by targeting IKK epsilon. Viruses (2019) 11(11). doi: 10.3390/v11111024 PMC6893776 31690057

[47] Bowen JR Quicke KM Maddur MS O'Neal JT McDonald CE Fedorova NB. Zika virus antagonizes type I interferon responses during infection of human dendritic cells. PloS Pathog (2017) 13(2):e1006164. doi: 10.1371/journal.ppat.1006164 28152048 PMC5289613

[48] Ank N West H Bartholdy C Eriksson K Thomsen AR Paludan SR. Lambda interferon (IFN-lambda), a type III IFN, is induced by viruses and IFNs and displays potent antiviral activity against select virus infections *in vivo* . J Virol (2006) 80(9):4501–9. doi: 10.1128/JVI.80.9.4501-4509.2006 PMC1472004 16611910

[49] Casazza RL Lazear HM Miner JJ. Protective and pathogenic effects of interferon signaling during pregnancy. Viral Immunol (2020) 33(1):3–11. doi: 10.1089/vim.2019.0076 31545139 PMC6978785

[50] Gao D Ciancanelli MJ Zhang P Harschnitz O Bondet V Hasek M. TLR3 controls constitutive IFN-beta antiviral immunity in human fibroblasts and cortical neurons. J Clin Invest (2021) 131(1). doi: 10.1172/JCI134529 PMC7773389 33393505

[51] Jagger BW Miner JJ Cao B Arora N Smith AM Kovacs A. Gestational stage and IFN-lambda signaling regulate ZIKV infection *In utero* . Cell Host Microbe (2017) 22(3):366–376 e3. doi: 10.1016/j.chom.2017.08.012 28910635 PMC5647680

[52] Patel MV Hopkins DC Barr FD Wira CR. Sex hormones and aging modulate interferon lambda 1 production and signaling by human uterine epithelial cells and fibroblasts. Front Immunol (2021) 12:718380. doi: 10.3389/fimmu.2021.718380 34630393 PMC8497887

[53] Jabrane-Ferrat N. Features of human decidual NK cells in healthy pregnancy and during viral infection. Front Immunol (2019) 10:1397. doi: 10.3389/fimmu.2019.01397 31379803 PMC6660262

[54] Dukhovny A Lamkiewicz K Chen Q Fricke M Jabrane-Ferrat N Marz M. A CRISPR activation screen identifies genes that protect against zika virus infection. J Virol (2019) 93(16). doi: 10.1128/JVI.00211-19 PMC6675891 31142663

[55] Chen Q Gouilly J Ferrat YJ Espino A Glaziou Q Cartron G. Metabolic reprogramming by zika virus provokes inflammation in human placenta. Nat Commun (2020) 11(1):2967. doi: 10.1038/s41467-020-16754-z 32528049 PMC7290035

[56] Meertens L Carnec X Lecoin MP Ramdasi R Guivel-Benhassine F Lew E. The TIM and TAM families of phosphatidylserine receptors mediate dengue virus entry. Cell Host Microbe (2012) 12(4):544–57. doi: 10.1016/j.chom.2012.08.009 PMC3572209 23084921

[57] Jabrane-Ferrat N El Costa H. Decidua basalis: An ex vivo model to study HIV-1 infection during pregnancy and beyond. Methods Mol Biol (2022) 2407:205–13. doi: 10.1007/978-1-0716-1871-4_15 34985667

