# Supplementary material for: The mechanisms underlying the immune control of Zika virus infection at the maternal-fetal interface
Source: Front Immunol. 2022 Nov 22;13:1000861. doi: 10.3389/fimmu.2022.1000861 (PMC9723234; doi:10.3389/fimmu.2022.1000861)
Supplement: Supplementary file 1 [file DataSheet_1.docx]

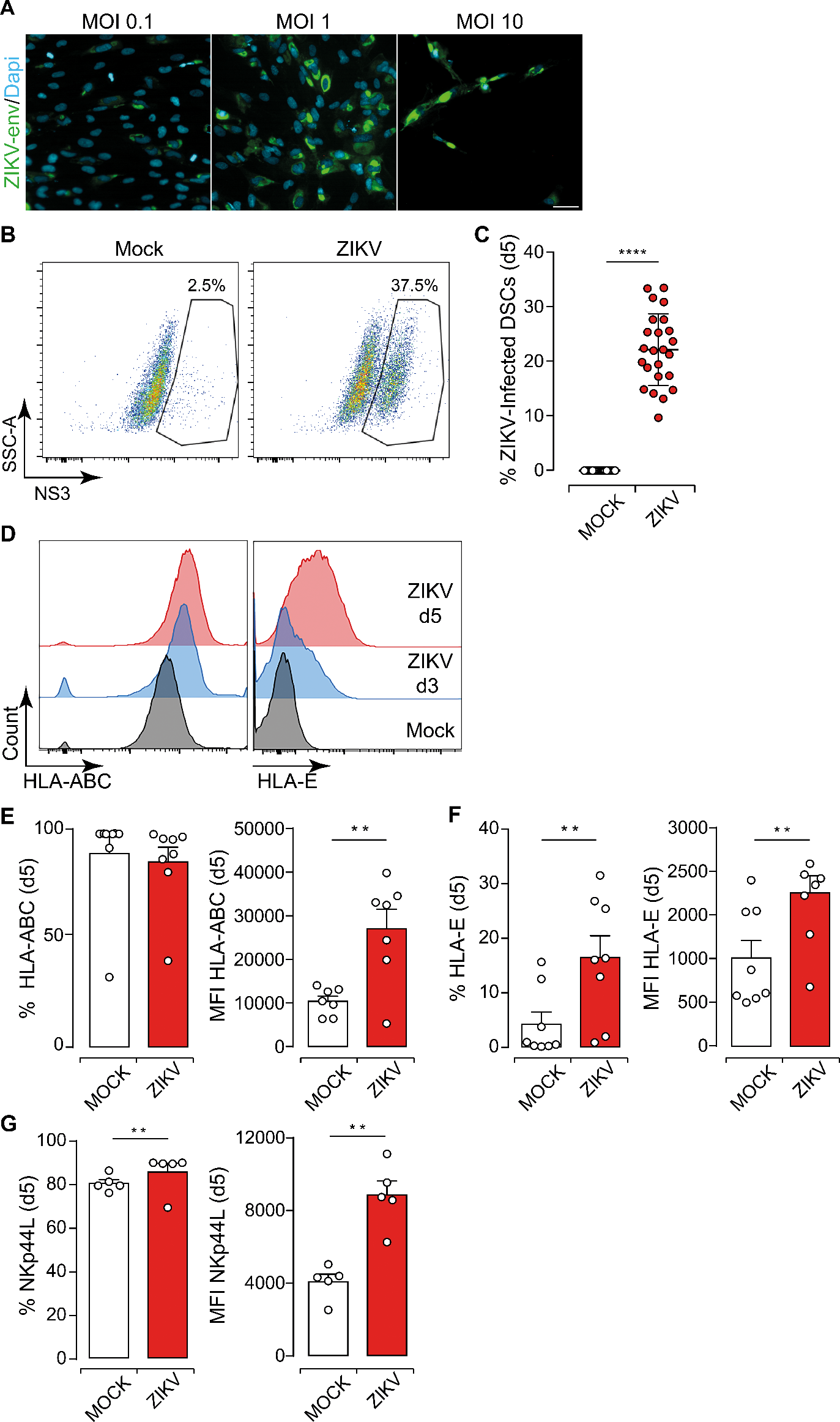


Figure S1

ZIKV Infection Modulates the Expression of the Classical and Non-Classical MHC and NKR Ligands. **(A)** DSCs were challenged with different MOI (0.1, 1 and 10) of ZIKV. Representative large field view of ZIKV-infected DSCs expressing the ZIKV-envelope protein at day 3 post-infection (3 dpi). **(B-C)** DSCs were challenged with ZIKV at a MOI of 1. ZIKV viral protein was determined at 5 dpi by flow cytometry using anti-NS3 antibody, followed by fluorochrome-conjugated secondary antibody staining. Gating strategies for the expression of ZIKV protein in uninfected (MOCK) and ZIKV infected DSCs (ZIKV) **(C)**. Percentage of Mock- (White dots), ZIKV-infected (Red dots) DSCs with intracellular expression of ZIKV protein **(D)**. Representative histograms of HLA expression in Mock and ZIKV infected DCSs at 3 dpi (d3) and 5 dpi (d5). **(E-G)** Cell surface expression of specific NK cell receptor ligands determined by FACS from MOCK- (white) and ZIKV-infected (red) DSCs. Percentage and mean fluorescence intensity (MFI) for the expression of HLA-ABC **(E)**, HLA-E molecules **(F)** and NKp44 ligands, NKp44L **(G)**. Data sets represent mean values ± SEM determined from at least five independent donors. *P* values are computed using paired two-tailed Student’s *t* test. **p<0.01, ****p<0.0001.


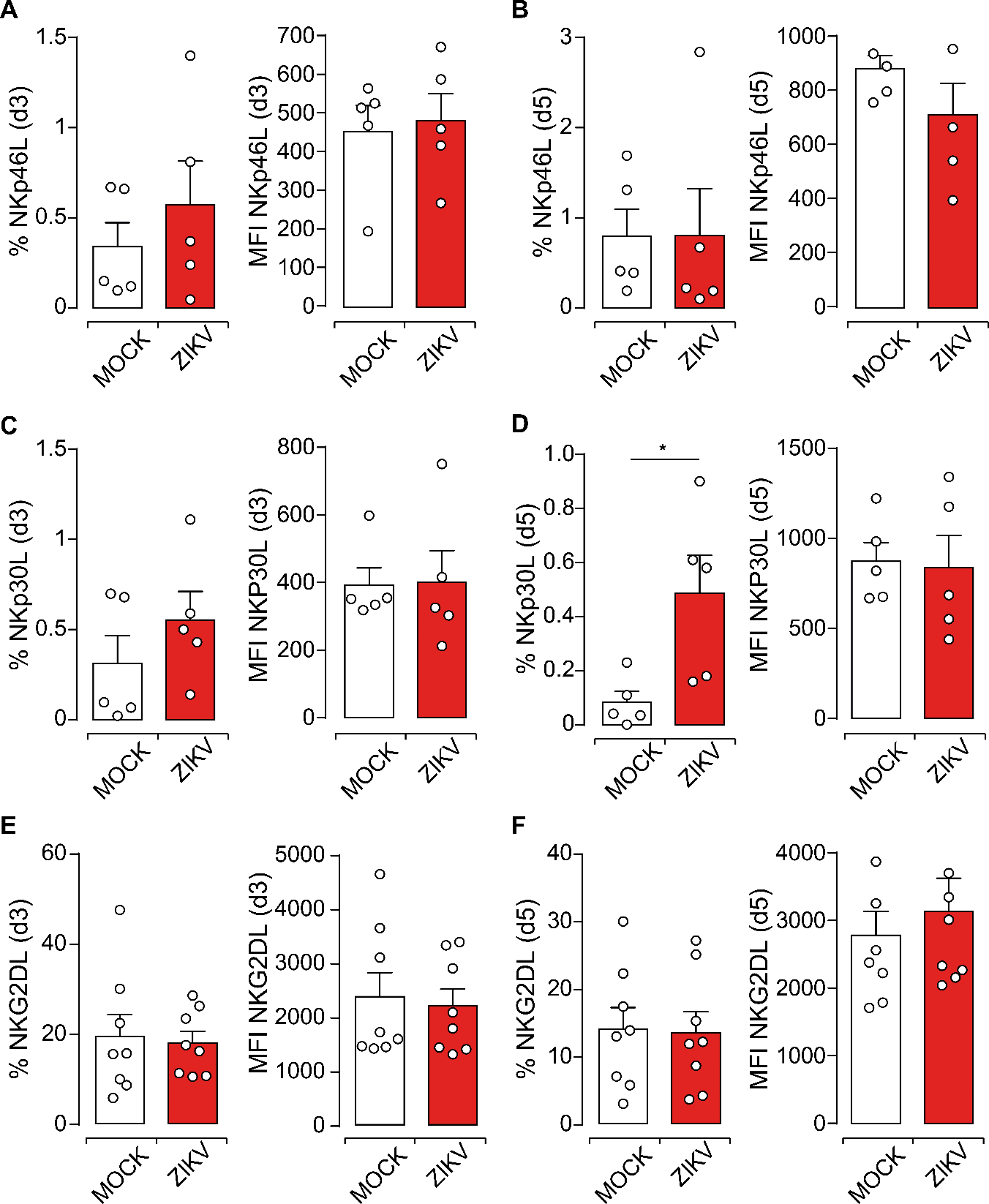


Figure S2

Modulation of the Expression of NKR Ligands by ZIKV Infection of Decidual Stroma Cells.

**(A-F).** Cell surface expression of specific NK cell receptor ligands at 3 and 5 dpi determined by FACS. MOCK-uninfected (white) and infected (red) DSCs. Percentage (%) and mean fluorescence (MFI) of the expression of NKp46L at 3dpi **(A)** and 5dpi **(B)**. Percentage (%) and mean fluorescence of the expression (MFI) of NKp30L at 3dpi (**c**) and 5dpi **(D)**. Percentage (%) and mean fluorescence (MFI) of the expression of NKG2D at 3dpi **(E)** and 5dpi **(F)**. Data sets represent mean values ±SEM determined from at least five independent donors. *P* values are computed using paired two-tailed Student’s *t*-test. p>0.05 are considered not significant.


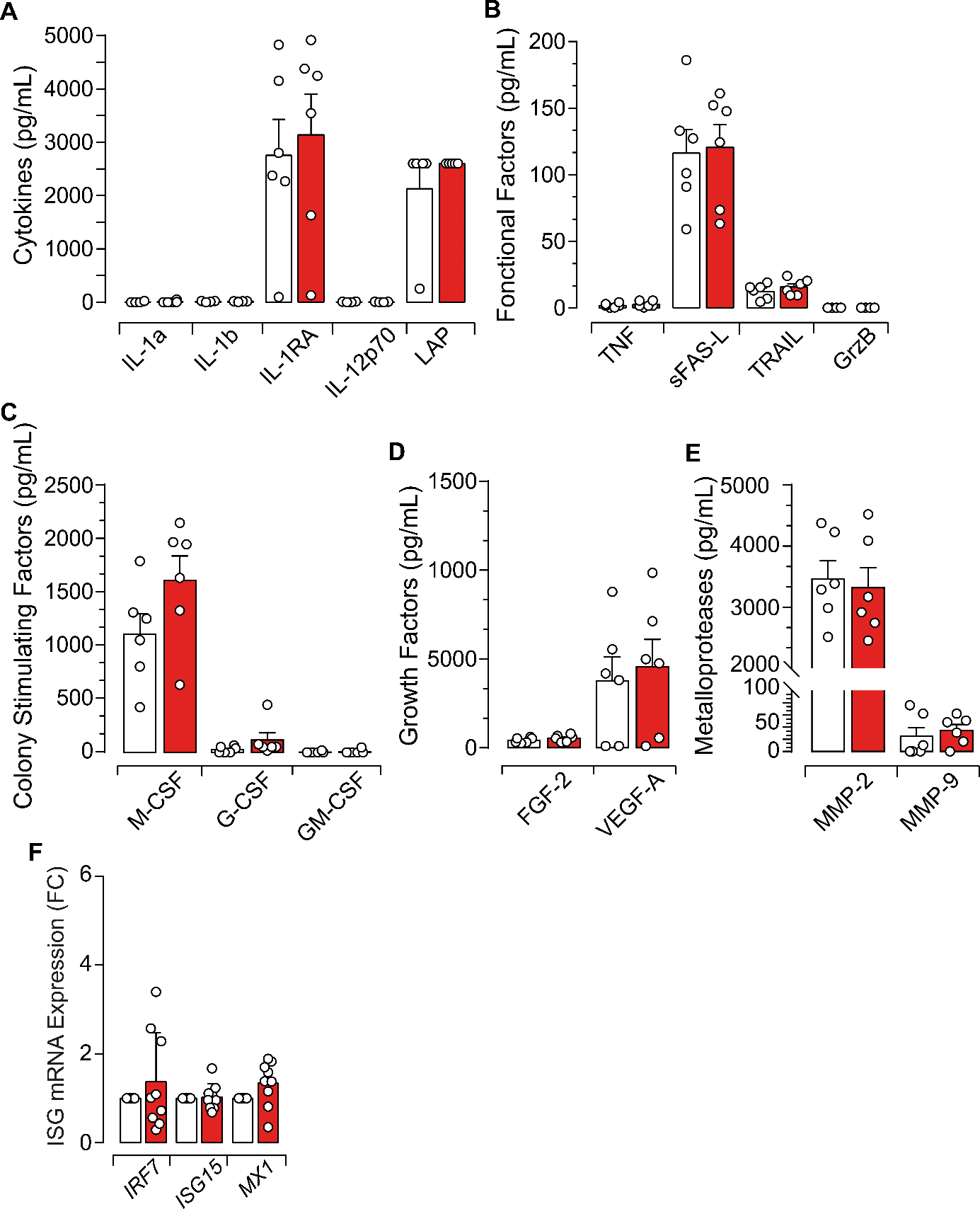


Figure S3

Modulation of DSC Secretome by ZIKV Infection**.** DSCs were infected with ZIKV at an MOI of 1. **(A)** Cytokines, **(B)** Functional factors, **(C)** Colony Stimulating Factors, **(D)** Growth Factors and **(E)** Metalloproteases quantified in the supernatants collected 48 hours post-viral challenge using a 42-multi-plexed cytokine assay. **(F)** *IRF7*, *ISG15* and *MX1* transcripts were quantified using qRT-PCR and specific primers. Representative histograms showing significant differences between secretions of uninfected (white bars) and ZIKV-infected DSCs (red bars) are presented. All data sets were normalized and presented as mean values ± SEM from at least six independent donors. Statistical comparisons were performed between uninfected and ZIKV-infected DSCs using paired two-tailed Student’s *t*-test.


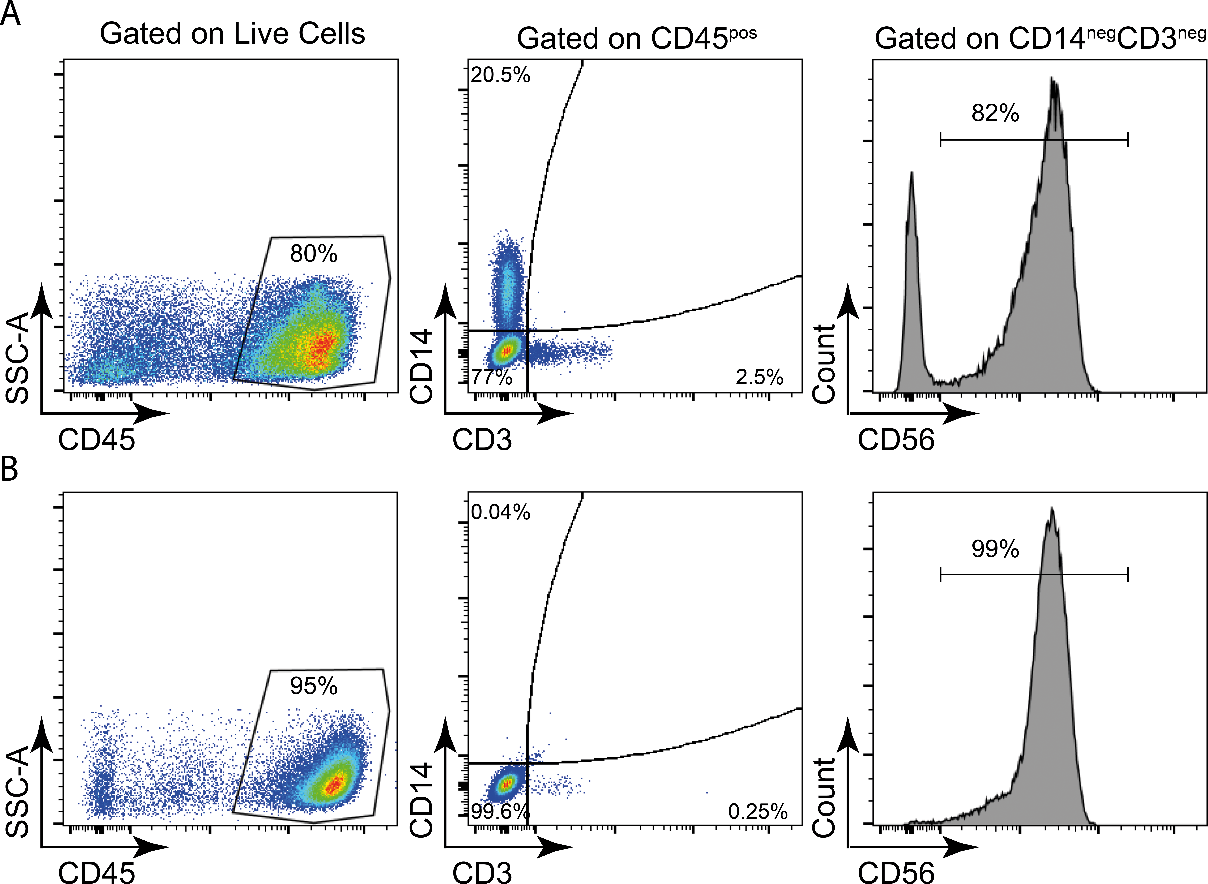


Figure S4

Gating Strategies for dNK Cell Isolation. dNK cells**.** Analysis of dNK cells using specific marker and flow cytometry. dNK cell are analyzed using specific antibodies before **(A)** and after **(B)** purification using MACS negative selection kit (Miltenyi Biotech). Cells were labeled with live-dead fixable dye and immunostained with cytochrome-conjugated anti-CD45, -CD3, -CD14 and -CD56 antibodies. Dot plots were gated on CD45^pos^ live cells. The CD3^neg^ and CD14^neg^ cells are then analysed for the expression of CD56 marker. dNK cells represented 77% of the CD45^pos^, CD3^neg^ and CD14^neg^ cells before purification **(A)** and 99% after purification **(B)**.
